# Factors associated with hospital service satisfaction in a sample of Arab subjects with schizophrenia

**DOI:** 10.1186/1472-6963-10-294

**Published:** 2010-10-27

**Authors:** Muhammad A Zahid, Jude U Ohaeri, Adel A Al-Zayed

**Affiliations:** 1 Department of Psychiatry, Faculty of Medicine, Kuwait University; P.O. Box 24923, Safat, 13110, Kuwait; 2 Department of Psychiatry, Psychological Medicine Hospital, Kuwait

## Abstract

**Background:**

Assessment of patients' satisfaction with health care services could help to identify the strengths and weaknesses of the system and provide guidance for further development. The study's objectives were to: (i) assess the pattern of satisfaction with hospital care for a sample of people with schizophrenia in Kuwait, using the Verona Service Satisfaction Scale (VSSS-EU); ii) compare the pattern of satisfaction with those of similar studies; and iii) assess the association of VSSS seven domains with a number of variables representing met and unmet needs for care, family caregiver burden, severity of psychopathology, level of psychosocial functioning, socio-demographic characteristics, psychological well-being and objective quality of life.

**Methods:**

Consecutive outpatients in stable condition and their family caregivers were interviewed with the VSSS-EU and measures of needs for care, caregiver burden, quality of life and psychopathology.

**Results:**

There were 130 patients (66.1%m, mean age 36.8). While over two-thirds expressed satisfaction with the domains of "overall satisfaction", "professionals' skills", "access", "efficacy", and "relatives' involvement", only about one-third were satisfied with the domains of "information" and "types of intervention". The later two domains were the areas in which European patients had better satisfaction than our patients, while our patients expressed better satisfaction than the Europeans in the domain of "relatives' involvement". In multiple regression analyses, self-esteem, positive and negative affect were the most important correlates of the domains of service satisfaction, while clinical severity, caregiver burden and health unmet needs for care played relatively minor roles.

**Conclusion:**

The noted differences and similarities with the international data, as well as the predictive power of self-esteem and affective state, support the impression that patients' attitudes towards psychiatric care involve a complex relationship between clinical, personal and socio-cultural characteristics; and that many of the factors that impact on satisfaction with service relate to individual psychological characteristics. The weaknesses in the system, highlighted by the pattern of responses of the participants, indicate possible gaps in the provision of comprehensive psychiatric care in the country and obviate the need for public mental health education and development of services to enhance the quality of care.

## Background

Assessment of patients' satisfaction with mental health services has become accepted by health planners as a measure of quality of care [1,2]. Knowledge of the pattern of service satisfaction could help to identify the strengths and weaknesses of services, and provide guidance for further development [3-5]. Despite the reservations in the literature about the capacity of patients with severe mental disorders to give reliable opinion about satisfaction with services, and the possibility of biased opinion because of a tendency towards "an agreeing response set" [6], it was found that, patient satisfaction, but not clinician or referrer satisfaction, was a more accurate indicator of quality of care than standard indicators [7].

A review of the literature showed that the factors associated with psychiatric service satisfaction could be classified into three broad groups, namely: institutional/treatment - related issues of quality of care; clinical severity and socio-demographic characteristics; and socio-cultural/psychological attributes of the patient [1,5,6]. In general, patients receiving care in community settings express higher satisfaction than those in hospital - based services [8], and service effectiveness is associated with higher satisfaction [9-11]. There have been fewer attempts to relate service satisfaction with other indices of quality of care, such as patients' unmet needs for care, quality of life (QOL) and family caregiver burden [1,4,12,13]. On the other hand, there are more reports indicating that dissatisfaction with service provided is associated with lower physical and mental health, severity of behavior problems, level of psychosocial functioning, and duration of illness [2,14-17]. However, there are conflicting findings about the relationship of socio-demographic characteristics with service satisfaction [16]. While most studies found no significant gender difference [1,17-20], others found that women had higher satisfaction scores than men [15,21]. Also, age was reported to be correlated with aspects of service satisfaction in most studies [18,19,21], but not in others [17]. Furthermore, there are conflicting reports about the role of socio-economic indices (such as education, money and housing) [17,22] and marital status [23]. With regard to psychological attributes, the most consistent findings are that, service satisfaction is significantly associated with subjective quality of life (QOL) [12,24,25]; and abnormal personality traits are negatively correlated with satisfaction [22,26].

Service satisfaction has usually been viewed as a multidimensional construct and has, therefore, been measured as such, in order to assess its various facets [5-7,16,18,19]. In a recent (2009) review of related instruments, it was found that, although there are currently many instruments, the methods used to generate them are heterogeneous, causing the content of questionnaires to vary; and reliability and validity were not systematically tested [5]. In addition, instruments have often been limited to a few broad items which require only one or two dimensions of mental health care [19]. In view of this situation, we chose the 54-item Verona Service Satisfaction Scale (VSSS -EU version) because it has received the most research attention in psychiatry, having been validated in the five-nation European Psychiatric Services: Input Linked to Outcome Domains and Needs (EPSILON) study [27]. Unlike other instruments that focus on specific services (e.g., inpatient or outpatient services) [5,6,16], the VSSS attempts a broad - based assessment. It covers seven broad domains, namely, "overall satisfaction" (3 items), "professionals' skills and behavior" (16 items), "information" (3 items), "access" (2 items), "efficacy" (8 items), "types of intervention" (17 items), and "relatives' involvement" (5 items). However, it has been noted that satisfaction with health care depends more on factors external to the health care system than on the experience of care as a patient [28]. Some of these external factors concern objective QOL and psychological well-being [1,16]. Hence, it is important to assess the relationship of indices of objective QOL (i.e., living situation, finances, and social relationships), and indices of psychological well -being, such as self-esteem, positive affect (i.e., positive mood states), and negative affect (i.e., negative mood states), with scores on domains of service satisfaction. First, variables related to self-esteem and affect (e.g., feeling happy with aspects of life) have been found to be associated with service satisfaction [1,4]. Second, reports from the field of positive psychology have shown the inter-relatedness of the constructs of satisfaction, self-esteem and positive/negative affect across cultures [29-32]. In view of the above relationships, our conceptual framework was Ware et al's [33] definition of service satisfaction, elaborated by Chow et al [18], as consisting of "satisfaction determinants" and "satisfaction components". Satisfaction and dissatisfaction indicate patients' judgment about the strengths and weaknesses, respectively, of the service [18]. The term "satisfaction components" refers to satisfaction with services actually provided (as in the domains of VSSS), while "satisfaction determinants" consist of patient's expectations, preferences, and individual characteristics (including socio-demographic and clinical characteristics, quality of life, self-esteem and ethnic origin) [18]. Accordingly, our analysis was geared towards assessing the association between indices of "satisfaction components" (i.e., VSSS domain scores) and indices of "satisfaction determinants", with a view to understanding the relationship between the strengths and weaknesses of the service, on the one hand, and individual characteristics, on the other hand.

The rationale for our study is that reports on service satisfaction from the Arab world concerned only studies of general/private hospitals and primary health care centers (PHC) [14,34-38], these studies did not link satisfaction components with satisfaction determinants, and there are no reports on psychiatric services. One Saudi Arabia primary health care study reported that 40% of the patients were dissatisfied [36], while two other reports found discrepancies between the reported satisfaction and the inadequacy of the resources [35,39]. Indeed, the ordinary expectation from psychological theory is that, despite differences in expectation [33], the all-pervading conservative religious culture, typical of what has been described as a "collectivistic society" [29,32], would tend to encourage people in these settings to express more satisfaction with services provided to them, in comparison with the "individualistic societies" of the western world [18,31].

In this study, we examined a wide variety of issues related to psychiatric hospital service satisfaction among Kuwaiti subjects with schizophrenia, with a view to contributing information on the factors associated with the strengths and weaknesses of the mental health care system in Kuwait. Taking a cue from the EPSILON study reports [12,40], we have done this by relating data on domains of satisfaction components (as in the VSSS-EU), with indices of satisfaction determinants, such as, socio-demographic and clinical characteristics, as well as broad indices of quality of care, namely, met/unmet needs for care as in the Camberwell Assessment of Needs (CAN) [41], and family caregiver burden as in the Involvement Evaluation Questionnaire (IEQ) [42].

The specific objectives of the study were to: (i) assess the pattern of satisfaction with psychiatric hospital care for a sample of people with schizophrenia in Kuwait, using the VSSS-EU, with a view to seeing the areas of the service that the patients were satisfied and dissatisfied with; ii) compare the pattern of patient satisfaction with hospital care of people with schizophrenia in Kuwait with those of similar studies [12,19]; and iii) assess the association of seven VSSS domains of service satisfaction with a number of variables representing met and unmet needs for care, family caregiver burden, severity of psychopathology, level of global psychosocial functioning, socio-demographic characteristics, psychological well-being and objective quality of life. These factors have been found to have variable predictive powers on service satisfaction [1,12,16,18,19].

Considering the nature of the Kuwaiti society [18,29-32] and the type of mental health care service (details provided below), we hypothesized that the patients would be generally satisfied with the service, especially those aspects of the service that relate to the strengths of the conservative culture, namely, the involvement of relatives. The differences in pattern of satisfaction with the international literature would be a reflection of differences in culture and type of service [12,18]. Furthermore, in multivariate analyses, service satisfaction would be significantly associated with socio-demographic characteristics and indices of quality of care, objective QOL, clinical severity and psychological well-being [1,18,19].

## Methods

### The setting

Kuwait is an Arab country, a city - state located in the Arabian Gulf. Of the total 3.4 million population, Kuwaiti nationals make up 1.1 million (2007 census). For Kuwaiti nationals, there is an effective national social welfare system. The country has a conservative Muslim culture, with traditional gender roles and sexual segregation, and the extended family system and family social support are the norms. According to psychological theory, these characteristics place Kuwait in the category of a so called "collectivistic society", which is said to have different patterns of satisfaction with the so called "individualistic societies" of the Western world [29-32].

The study was carried out at the Psychological Medicine Hospital, the only facility of its kind in Kuwait. The hospital is housed in a modern, spacious, well equipped set of buildings, and consists of 691 in-patient beds. There are no community - based mental health care services. All services provided to Kuwaiti nationals are free - of - charge. Health care delivery is sectorized (i.e., in catchment areas). There are five general adult psychiatric catchment area units. Patients involved in this study belonged to the catchment area of one of us (MAZ). In view of the uniform socio-economic and cultural circumstances in such a small country, there is no reason to believe that the characteristics of these patients are different from those of patients from the other catchment areas.

### Subjects

The participants consisted of consecutive attendees at the unit, who fulfilled the study's inclusion criteria. We sought to have participants that were comparable with those of studies that have used the same instruments to study patients of comparable diagnostic groups [12,19]. Hence, the participants had recently been discharged from admission, had attended follow - up clinic appointment, had been ill for at least one year, were aged less than 65 years, were literate in Arabic, could independently provide informed consent to participate, and were in stable clinical condition. In addition, they were accompanied by family caregivers who lived with them. All patients had a stable (at least one year) case note diagnosis of schizophrenia, which was verified by the administration of the ICD-10 Symptom Checklist as in the Schedule for Clinical Assessment in Neuropsychiatry [43].

Of the 146 patient - family caregiver dyads that fulfilled the inclusion criteria during the study period, 16 did not complete the full interviews because they failed to attend follow-up appointments. The 16 patients who did not complete the interviews consisted of 13 men and three women, mean age 44.1(13.1). This report concerns the 130 patients who completed the interviews (68.5% men, aged 14-61 yrs, mean 36.8, SD 10)

Over 80% (of 130 full participants) had at least high school education, 35 (26.9%) were currently married, and 95.5% were living together with either their spouses or families of origin. Their global level of psychosocial functioning was average (as assessed by the Global Assessment of Functioning - GAF score = 50.2), and the mean BPRS (psychopathology) (18 - item) score of 44.4 indicated that they were clinically "moderately ill" [44].

### Ethical approval

Ethical approval for the work was obtained from the Research and Ethical Committee of the Faculty of Medicine, Kuwait University. Patients and family caregivers gave verbal informed consent after the objectives of the study had been explained to them. They were duly informed that there would be no negative consequences for declining to participate, and that they were within their rights to refuse to participate. As is well known in this culture for such non-invasive studies [25], all families approached freely consented to participate.

### Assessment instruments

The subjects were assessed with the instruments used for the EPSILON study, namely: the European versions of: (i) the Verona Service Satisfaction Scale [27]; (ii) the Camberwell Assessment of Need (CAN-EU) [41]; (iii) the Involvement Evaluation Questionnaire - for the relatives (IEQ-EU) [42]; and (iv) the Lancashire Quality of Life Profile (LQoLP-EU) [45]. All these instruments were slightly modified to suit the Kuwaiti situation and translated into Arabic by the method of back - translation. Psychopathology was assessed with the following instruments: 14 items of the ICD-10 Symptom Checklist [43], and the 24-item version of the Brief Psychiatric Rating Scale (BPRS) [46]. Global level of psychosocial functioning was assessed with the Global Assessment of Functioning scale (GAF) [46].

We obtained all the assessment instruments of the EPSILON study from the authors [46], who approved our Arabic translation.

Only the VSSS-EU will be described in detail here because it is the focus of this report. Details about the reliability indices and contents of the other questionnaires have been presented elsewhere [25]. In brief, they were found to be applicable in Kuwait, and both the inter-rater reliability tests (for the two raters, using intra-class correlation coefficient) and internal consistency for all participants (using Cronbach's alpha) were all adequate (i.e., > 0.7).

### The Verona Service Satisfaction Scale (VSSS-EU)

The VSSS-EU [27] is a 54 - item self - administered instrument that consists of seven domains (already highlighted). The response options are: "terrible", "mostly dissatisfied", "mixed", "mostly satisfied", and "excellent", on a scale of 1-5; so that higher subscale/domain scores indicate better satisfaction.

The most important subscale is the "overall satisfaction", as it gives a global impression of satisfaction with services by assessing the "amount of help received", "kind of services" and "overall satisfaction". The subscale on professionals (we focused only on the attitudes towards psychiatrists) assesses perceptions of the competence and manners of the professionals. The information subscale is concerned with explanations given to the patient about the services offered. Access subscale deals with the physical layout and costs of the service. The efficacy domain concerns the perceived effectiveness of the service in helping the patient in the clinical and psychosocial areas of living. The subscale on "types of intervention" assesses the service's response to crises situations, as well as the provision of opportunities for activities outside the hospital's premises. The subscale on "relatives' involvement" concerns perceptions of how much the professionals have been able to help the family caregiver to cope with the patient's problems.

The subscale scores are computed by the average of the total for the relevant subscale items [27]. The recommended cut-off score for dissatisfaction for each subscale is < 3.5 [12]. The VSSS has been validated in various international studies [47,48].

It required minor modifications to make it suitable for our setting. Thus, six items of the service provision section were not included in our analysis for the following reasons: (i) compulsory treatment: this service is provided only for forensic cases; (ii) sheltered accommodation is not available in Kuwait; (iii) leisure activities outside the hospital is provided occasionally for only inpatients; (iv) sheltered work is not available for patients; (v) help from the hospital to find open employment is not provided; (vi) the leisure activities (e.g., sports) are within the hospital. As a result, the subscale for "types of interventions" consisted of 11 items rather than 17, and the total number of items we used was 48. In the Danish study of patients with affective disorders, these were also among the items removed from the VSSS because they were found to be irrelevant for such patients [19].

It is recommended that the VSSS should be self-administered [27]. However, because of the length of the questionnaire and the need to ensure that the participants understood the questions, all assessments were interview-based, and carried out in Arabic by two Arab psychiatric registrars. But the participants were duly assured that this was a research activity, their responses would be recorded anonymously, and that their responses would not lead to any adverse consequences. The reliability coefficients were quite satisfactory. First, the inter-rater reliability for the two interviewers, using the responses of 14 subjects, who did not take part in the main study, was as follows: intra-class correlation coefficient (ICC) for the items (i.e., 48 + 48) was 0.94 (95% C.I. = 0.86 - 0.99). Kendall's tau correlation (r) for each pair of subscale scores (i.e., for both interviewers) was as follows: intervention: 0.85; information: 0.82; access: 0.60; overall satisfaction: 0.43; efficacy: 0.88; relatives' involvement: 0.76; professionals' skills: 0.67; total scores: 0.78. We used Kendall's tau because it is more conservative than Pearson's correlation as it takes ties into consideration. For the responses of the participants of the main study (N = 130, number of items = 48), the internal consistency (Cronbach's alpha) was 0.97. The internal consistency values for each of the subscales are presented in Table 1 for the main study's participants.

**Table 1:** VSSS domain scores, internal consistency, and proportion of subjects satisfied with domains: compared with other studies

VSSS domain/subscale	No. of items	Reliability: Cronbach's Alpha (N = 130)	Unadjusted mean score (SD): N = 130	Adjusted mean score* (SE) N = 111	(%) dissatisfied with domain i.e. unadjusted score < 3.5 **	(%) satisfied with domain i.e. unadjusted score >/= 3.5	Unadjusted mean (SD) scores EPSILON: Pooled data N = 399***	Danish study of subjects with mood disorders: Aged < 40 yrs/Aged ≥40 yrs Mean (SD)****
Overall satisfaction	3	0.81	4.04(0.71)	4.07(0.06)	28(21.5)	102(69.4)	3.83(0.79)	3.63(1.06)/ 4.00(1.00)
Professional skills	16	0.94	3.85(0.66)	3.89(0.06)	35(26.9)	95(73.1)	3.88(0.57)	3.60(0.98)/ 3.98(0.84)
Information	3	0.66	3.26(0.81)	3.26(0.08)	89(68.5)	41(31.5)	3.39(0.93)	3.44(1.10)/ 3.76(1.08)
Access	2	-	3.59(0.87)	3.58(0.08)	44(33.8)	86(66.2)	3.83(0.73)	3.41(1.46)/ 3.56(1.33)
Efficacy	8	0.91	3.83(0.68)	3.87(0.06)	31(23.8)	99(76.2)	3.56(0.74)	3.38(1.08)/ 3.65(1.00)
Types of Intervention	11*****	0.75	3.39(0.45)	3.42(0.04)	87(66.9)	43(33.1)	3.64(0.42)	3.54(0.61)/ 3.85(0.93)
Relatives' involvement	5	0.88	3.79(0.68)	3.82(0.06)	36(27.7)	94(72.3)	3.39(0.96)	2.92(1.26)/ 3.19(1.34)
Total VSSS score	48	0.97	3.68(0.54)	3.71(0.05)	42(32.3)	88(67.7)	3.70(0.50)	3.40(0.98)/ 3.73(0.93)

*Adjusted for sex, age, education, marital status, family income, duration of illness, Brief Psychiatric Rating Scale score, and Global Assessment of Functioning score. N is < 130 because of missing values.

** Using the recommended cut-off score (< 3.5) for dissatisfaction/satisfaction (Ruggeri et al., 2003) [12].

*** Ruggeri M et al 2000 [27]; **** Kessing LV et al 2006 [19]; ***** Six items of the service provision section were not included (see text for details)

### Patients' met/unmet needs

The CAN-EU [41], an interviewer-administered instrument, assesses patients' needs as perceived by them (as users) and the staff who have knowledge of them. It comprises 22 items of met and unmet needs (user and staff perceptions). The scores of the 22 items are gathered into five groups of met and unmet needs, namely: basic (3 items), health (7 items), social (3 items), functioning (5 items), and service (4 items). We used only the data for user (i.e., patient) for this report.

### Caregiving consequences

The IEQ-EU [42] is an 81-item self-administered instrument that measures the consequences of psychiatric disorders for relatives of patients in the past four weeks. The 31 items on caregiving consequences are grouped into four scales, namely, tension, worrying, urging, and supervision. The 12 - item Goldberg's General Health Questionnaire [49] is included.

### Lancashire Quality of Life Profile (LQoLP-EU)

The LQoLP-EU [45], a structured interviewer - administered instrument for measuring the health and welfare of people with mental disorders, is one of the most widely used instruments for the assessment of QOL in schizophrenia research [50]. It combines objective, factual, material related to several different life domains (i.e., objective QOL indicators) with subjective satisfaction with those domains (i.e., subjective QOL indicators). This report concerns only the objective QOL indicators.

The objective components are evaluated on a scale of: Yes/No/Don't know. The objective QOL items involved in this report are: (i) participation in leisure activities; (ii) finances; (iii) living situation; (iv) family relations; and (v) social relations.

The questionnaire allows for the assessment of the following additional areas: (a) five positive items for positive affect and five negative items for negative affect from the Bradburn Scale [44]; and (b) the 10 - item Rosenberg Self-esteem scale [51].

### Data collection procedure

At the preliminary stage of the study, the research team scrutinized the questionnaires for appropriateness of content in the Kuwaiti setting. After slight modifications, two native Arabs, who are fluent in English, jointly produced the Arabic translations of the instruments by the method of back - translation.

Thereafter, one of us (MAZ), an experienced British - trained psychiatrist, trained two Arab psychiatric registrars in the use of the questionnaires.

Assessments generally took place at intervals over a period of one week, to suit the convenience of the families. The BPRS and GAF were administered first, so that the ratings would not be influenced by performance or responses on subsequent measures [52].

### Data analysis

Data were analyzed by the SPSS 15 (SPSS Inc., Chicago, Illinois). We used parametric statistics because the VSSS data were fairly normally distributed.

### Dependent and independent variables

Following our conceptual framework [18,33], the seven VSSS subscales scores and total score were the dependent variables for all subsequent analyses. The scores from all the other questionnaires constituted the independent variables. Thus, the five groups of met and unmet needs were computed from the items of the CAN-EU. From the items of the IEQ-EU, we computed the four scales of caregiving consequences and the total GHQ-12 score. From the LQoLP-EU, we computed the scores for positive/negative self-esteem, and positive/negative affect. The following psychopathological scores were computed: (a) the scores of the ICD-10 symptom checklist were grouped into positive and negative symptoms; and (b) total BPRS score.

### Pattern of VSSS subscale scores and international comparisons

For the first objective, we used frequency counts and mean scores to examine the pattern of VSSS subscale scores. We examined the relative strengths and weaknesses of the service by assessing significant differences between domain scores using paired t-test. For the second objective, quantitative differences between our domain scores and those of similar reports [19,27] were analyzed by standardized effect size calculations.

### Associations of VSSS subscale scores

For the third objective, we used t-tests, one-way analysis of variance (ANOVA) and Pearson's correlations, to examine the univariate association between the VSSS subscale scores and each of the independent variables. In view of the results of the univariate analyses, the association of VSSS subscale scores with all the independent variables was assessed by step-wise multiple regression analyses. Based on the pattern of their associations from the univariate analyses, the independent variables were entered in six steps, thus: Block 1: socio-demographics; block 2: clinical characteristics highlighted above; block 3: positive affect, negative affect, positive self-esteem, negative self-esteem, total self-esteem; block 4: met needs' subscales and total for user; block 5: unmet needs and total for user; block 6: IEQ subscale scores. For the multiple regression data, multi-collinearity was assessed by the values of "tolerance" (cut-off score </= 0.2) and variance inflation factor (VIF - cut-off score > 4.0).

All tests were two-tailed. A Bonferroni correction (P = 0.01) was applied for multiple tests; otherwise, the level of statistical significance was set at P < 0.05

## Results

### VSSS subscale scores

Using a cut-off score of < 3.5, we found that the participants were generally satisfied with the services, because at least two-thirds expressed overall satisfaction, as well as satisfaction with the other domains; except "information" and "types of intervention" where less than one-third expressed satisfaction. Accordingly, the least scores were for "information" and "types of intervention". The subscale score for "information" was significantly lower than those for "professionals' skills", "access", "relatives' involvement", "efficacy", and "overall satisfaction" (paired t ranged from 3.7 - 10.1, df = 129, P < 0.0001). Similarly, the "types of intervention" score was significantly lower than those for "professionals' skills", "efficacy" and "overall satisfaction" (t = 10.1, P < 0.0001). In other words, the patients indicated that the weakest aspects of the service were in the domains of "information" and "types of intervention".

### Comparison with similar data

We could only compare our mean scores with the EPSILON data for unadjusted scores, because the values for standard deviation were presented for only the unadjusted scores [27]. Also, we could not compute significant differences with the Danish report [19] because they did not present details for number of subjects by age group. The highlights are as follows (Table 1):

(i) Our VSSS subscale scores were either similar to, or not significantly different from the EPSILON pooled data (N = 399) for "professionals' skills", "information" and total scores (P > 0.05). Our subscale scores were significantly higher than the EPSILON pooled data for "overall satisfaction" (E.S: 0.27: 0.07 - 0.47); "efficacy" (0.37: 0.17 - 0.57); and "relatives' involvement" (0.44: 0.24 - 0.64). The EPSILON pooled data for "types of interventions" was significantly higher than the Kuwaiti score (0.58: 0.38 - 0.78).

(ii) The Kuwaiti subscale scores for "information" and "types of intervention" tended to be lower than those from the five EPSILON sites. For "information", this trend reached significance for Amsterdam (0.50: 0.19 - 0.82), Copenhagen, and Verona (P < 0.001)

When examined from the perspective of percentage of people with dissatisfaction (i.e., score < 3.5 for each subscale) [12], we found that (see Table 1), while 21.5% of Kuwaiti participants were judged to be dissatisfied for the "overall satisfaction" subscale, the range for the EPSILON sites was 26.0% to 42.2% for four sites, and 20.6% for Verona. Judging by the VSSS total score, the Kuwaiti dissatisfaction rate of 32.3% was at the middle of the range for the EPSILON sites (from 19.6% for Copenhagen to 60.2% for London). For the Danish report [19], although the Kuwaiti subscale scores were numerically similar to the Danes aged > 40 years, the Kuwaiti scores were higher for "relatives' involvement" and "efficacy"; and lower for "information" and "types of intervention".

### Univariate associations of VSSS subscale scores

(i) T-tests and one-way ANOVA:

There was a tendency for women to have higher domain satisfaction scores than men. While this trend seemed to reach significance only for "overall satisfaction" (t = 2.9, df = 128, P < 0.02), it was not up to our Bonferroni correction threshold. The tendency for those who were divorced to have higher satisfaction scores than those who were married, reached significance for "overall satisfaction" (F = 5.2, df = 2/127, P < 0.007).

(ii) Correlation analyses: After Bonferroni correction, age was significantly correlated only with "types of intervention" (r = 0.22, P < 0.01). Years of education was not significantly correlated with satisfaction (P > 0.05). Of the psychopathological indices, the BPRS scores were significantly associated with "relatives' involvement" (r = 0.22, P < 0.01). For level of psychosocial functioning, the GAF score was significantly associated with "types of intervention" (r = -0.23, P < 0.008).

(iii) With further reference to our third objective, VSSS subscale scores were not significantly correlated with any of the subscales of the CAN and IEQ, as well as caregiver GHQ-12 (P > 0.05).

(iv) However, self-esteem was significantly correlated with a number of the VSSS subscale scores, viz: (a) "professionals' skills": with self-esteem (r = 0.3, P < 0.001); (b) "information": with negative self-esteem (r = -0.21, P < 0.015); (c) "efficacy" with: self-esteem (r = 0.31, P < 0.001); (d) "types of intervention": with negative self-esteem (r = -0.19, P < 0.026); and (e) total VSSS score: with self-esteem (r = 0.28, P < 0.0001). The tendency for VSSS subscale scores to be associated with negative/positive affect (P < 0.02) did not meet the Bonferroni correction criterion.

(v) Of the objective QOL indices, those who were satisfied with their living condition (N = 31/77) had significantly higher satisfaction scores in all the VSSS subscales, except "information" and "types of intervention" (t ranged from 2.0 to 3.1, df = 106, P < 0.002). The relationship between VSSS subscale scores and other objective QOL indices did not meet the Bonferroni correction threshold. Hence, those who claimed to have enough money to enjoy themselves (N = 54/60), tended to have significantly higher satisfaction scores in all VSSS subscales (t ranged from 1.97 to 2.4, P < 0.03).

### Multiple regression analyses (Table 2)

**Table 2:** Association of VSSS subscales with socio-demographic, clinical variables, needs for care, caregiver burden, affect and self-esteem

Dependent variables: VSSS Subscales	Independent variables*	Variance (%)	Standard beta	T value	P value	Tolerance **	VIF **
Total VSSS score	Self-esteem Positive affect	15.5 5.0 (total: 20.5)	0.31 0.24	3.1 2.4	0.003 0.02	0.88 0.88	1.14 1.14
Overall SS	Positive self-esteem Negative affect	11.1 6.0 (total: 17.1)	0.36 0.24	3.8 2.6	0.0001 0.01	0.99 0.99	1.01 1.01
Professionals' skills	Self-esteem	15.6	0.39	4.1	0.0001	1.0	1.00
Information	Negative self-esteem Health unmet need: user	7.1 5.9 (total: 13.0)	-0.26 0.24	2.7 2.5	0.01 0.02	0.99 0.99	1.00 1.00
Access	-	-	-	-	-	-	-
Efficacy	Positive self-esteem Urge IEQ caregiver	18.9 3.8 (total: 22.7)	0.42 0.19	4.6 2.1	0.0001 0.04	0.99 0.99	1.00 1.00
Types of interventions	Positive symptoms: ICD 10 Positive affect Health unmet need user	5.5 4.7 4.1 (total: 14.3)	0.21 0.18 0.21	2.1 1.8 2.1	0.04 0.08 0.04	0.98 0.95 0.97	1.00 1.10 1.03
Relatives'involvement	BPRS score Self-esteem	4.3 6.5 (total: 10.9)	0.18 0.26	1.8 2.6	0.08 0.01	0.99 0.99	1.02 1.02

*Independent variables entered: Block 1: socio-demographics; block 2: clinical variables: duration illness, BPRS, GAF, number of ICD-10 checklist positive and negative symptoms; block 3: positive affect, negative affect, positive self-esteem, negative self-esteem, total self-esteem; block 4: met needs subscales and total for user; block 5: unmet needs and total for user; block 6: IEQ subscale scores.

** Multicollinearity values showed no significant multicollinearity: cut-off values are: Tolerance: ≤ 0.2; VIF: > 4

When the socio-demographic and clinical characteristics, along with the subscale scores for the CAN and IEQ, as well as positive/negative self-esteem and positive/negative affect were entered in step-wise regression analyses as independent variables, the following relationships with the VSSS subscale scores emerged (Table 2):

(i) self-esteem and affective state were the most frequent and important associations of the VSSS subscale scores. Self-esteem was the more important of the two, being significantly associated with all the VSSS subscales, except "access". Self-esteem and affective state were the only significant associations of the scores for total VSSS, "overall satisfaction" and "professionals' skills". However, self-esteem and affect accounted for only 15.6% to 20.5% of the variance explained in the three VSSS subscales.

(ii) Of the clinical characteristics, only the BPRS score (4.3% of variance for "relatives' involvement"), and ICD-10 positive symptoms (5.5% of the variance for "types of intervention") entered the equations.

(iii) Of the CAN subscales, only the health unmet need entered the equations, being significantly associated with VSSS "information" (5.9% of variance) and "types of intervention" (4.1% of variance).

(iv) Of the family caregiver burden subscales, only the "urge" IEQ domain entered the equations, accounting for 3.8% of the variance for VSSS "efficacy".

## Discussion

Using the responses of 130 Kuwaitis in stable condition with schizophrenia, we assessed the pattern of satisfaction with the national psychiatric service, in comparison with the international data, and examined the factors associated with satisfaction in the seven domains of the VSSS. Although the participants generally expressed satisfaction with the service, their responses indicated that the system was rather weak in the areas of information provided to users and the range of available interventions. However, the system was judged to have strengths in the behavior of professionals, the effectiveness of treatment and in the way that family caregivers were assisted.

### Comparison with the international data

Our finding of general satisfaction with the service is in line with those of most surveys [18,29,53], including the Arab world [39,54]. For instance, in all but five of the 21 countries involved in the World Health Survey 2003, more than half of the respondents reported feeling "very satisfied" or "fairly satisfied" with the services [28]. Interestingly, our subjects' perceived strengths and weaknesses of the service seemed to define both the similarities and differences between our results and those of the others [12,19], and indicate the possible influence of socio-cultural circumstance and type of service in judgments of satisfaction with service [55]. For example, while the European patients were less satisfied with the involvement of relatives in their care, this was a particular area of strength for our service. This is consequent on the fact that in a conservative culture, the extended family system makes more relatives available for the care of patients. Other reports from Europe have indicated dissatisfaction in the domain of relatives' involvement [19]. On the other hand, the general pattern of satisfaction with service in our hospital-based service had similarities with both community- and hospital-based services in the five European countries [12,27]. This can be accounted for by the small population and size of Kuwait, the easy accessibility of the service to the population, and the fact that the service is provided free-of-charge. One area of similarity is the fact that patients in both studies were least satisfied with the information provided for their care. This finding has been widely reported [6,21,26].

The dissatisfaction that our patients expressed with the domains of information and types of intervention is in line with our ordinary experience in Kuwait, where there is no program of public mental health education and no community - based services. Our findings indicate that the assessed services need to be developed since they were not perceived as satisfactory.

### Factors associated with service satisfaction

The results of our analyses show the salience of context in determining the factors associated with service satisfaction. First, sex was only a significant variable for overall satisfaction in univariate analysis, but was not significant in multivariate contexts [19,20]. Second, while age was associated with a number of VSSS subscale scores in univariate analyses, it was not a significant predictor of any subscale score in the larger multivariate context (Table 2). This is similar to the results from a Saudi Arabia study of PHC attendees [35].

In the large multivariate context, the most frequent significant associations of VSSS subscale scores were factors related to psychological well-being, namely, self-esteem and affective state, a finding which other authors have stressed [12,26,28]. In a Netherlands' study, it was found that variations in satisfaction were mostly attributable to variations in individual patient characteristics [56]. The implication of this finding is that the service should be developed with the aim to meet the needs and demands of people with different characteristics.

The association of BPRS score with "relatives' involvement" (Table 2) is noteworthy because it supports the impression that the most important contributor to caregiver burden is severity of psychopathology [57]. Equally noteworthy is the contribution of unmet needs for care in the health domain of the CAN, thus showing the salience of meeting patients' expectations in judgments of service satisfaction [58].

### Limitations and strengths

The findings are not generalisable, because the participants needed to fulfill several inclusion criteria, the patients were from one catchment area, and the study was cross-sectional [59]. In addition, we could not assess the full range of types of intervention as six of them are not available in our service. Assessment of service satisfaction by questionnaires has been criticized on the grounds that expressions of "satisfaction" by this method could hide a variety of negative experiences [53,60]. However, quantitative assessment of service satisfaction has been found to be reliable and valid [27,47,48,61]. Although the assessment was interview-based, the interviewers were junior staff, not authority figures whose presence could predispose the patients to make favorable ratings [6]. In support of our methodology is the high score of the reliability indices (Table 1). Furthermore, their pattern of satisfaction was in line with our reality experience and with the international reports.

## Conclusion

Our findings support the impression that patients' attitudes towards psychiatric care involve a complex relationship between clinical and socio-cultural characteristics [1,28,55]. Furthermore, the predictive power of self-esteem and affective state is in line with cross-national data from the field of positive psychology [27]. Since the patients indicated that the weaknesses of the service were mainly in the domains of "information" and "types of intervention", this suggests that there are gaps in the provision of comprehensive psychiatric care in the country, and obviates the need for public mental health education and other services perceived as not being satisfactory, in order to enhance the quality of care.

## Competing interests

The authors declare that they have no competing interests.

## Authors' contributions

MAA and JUO designed the study, analyzed the data and prepared the manuscript. MAA supervised data collection. AAA played an invaluable role in data collection. All the authors read the manuscript and approved it.

## Pre-publication history

The pre-publication history for this paper can be accessed here:

http://www.biomedcentral.com/1472-6963/10/294/prepub
